# The Role of Gut Microbiota in Pediatric Obesity and Metabolic Disorders: Insights from a Comprehensive Review

**DOI:** 10.3390/nu17111883

**Published:** 2025-05-30

**Authors:** Ana Maria Koller, Maria Oana Săsăran, Cristina Oana Mărginean

**Affiliations:** 1 Doctoral School, “George Emil Palade” University of Medicine, Pharmacy, Science, and Technology of Targu Mures, Gheorghe Marinescu Street No 38, 540136 Targu Mures, Romania; kolleranamaria@gmail.com; 2 Department of Pediatrics 3, “George Emil Palade” University of Medicine, Pharmacy, Science, and Technology of Targu Mures, Gheorghe Marinescu Street No 38, 540136 Targu Mures, Romania; 3 Department of Pediatrics 1, “George Emil Palade” University of Medicine, Pharmacy, Science, and Technology of Targu Mures, Gheorghe Marinescu Street No 38, 540136 Targu Mures, Romania; marginean.oana@gmail.com

**Keywords:** pediatric obesity, gut microbial composition, inflammatory and metabolic outcomes, dietary interventions, microbiota-modifying strategies

## Abstract

**Background:** Pediatric obesity represents a multifactorial condition in which gut microbiota dysbiosis, low-grade systemic inflammation, and metabolic dysfunction are intricately connected. **Objectives:** This systematic review sought to evaluate and integrate current findings regarding the interactions between gut microbial composition, dietary influences, inflammatory status, and metabolic outcomes in obese pediatric populations. **Methods:** A comprehensive search of PubMed, Scopus, and Web of Science databases was conducted for studies published from January 2010 onward. Eligible studies comprised randomized controlled trials, and cohort, cross-sectional, and longitudinal designs involving individuals aged ≤18 years. Study quality was appraised using the NIH Study Quality Assessment Tool. **Results:** Sixteen studies fulfilled the inclusion criteria. Dysbiosis was consistently observed among obese children, characterized by alterations in microbial diversity and abundance associated with increased inflammation and adverse metabolic profiles. Dietary interventions, notably symbiotic supplementation and adherence to Mediterranean diet patterns, were associated with favorable modulation of gut microbiota and inflammatory parameters. The majority of studies demonstrated high methodological quality, although minor observational limitations were noted. **Conclusions:** Gut microbiota dysregulation plays a central role in the development of metabolic and inflammatory complications associated with pediatric obesity. Although dietary and microbiota-modifying strategies show therapeutic promise, their effectiveness must be substantiated through robust, long-term studies.

## 1. Introduction

The global prevalence of obesity has risen sharply in recent decades. By 2016, approximately 1.9 billion adults were overweight and 650 million were obese, with 39 million children under five projected to be affected by 2020 [1]. Obesity is associated with diabetes, stroke, metabolic syndrome, cancer, and mental health disorders, emphasizing the urgent need for effective management strategies [2].

The gut microbiota (GM) has emerged as a critical player in maintaining human health and preventing disease [3]. Comprising approximately 10^14^ microorganisms, including bacteria, viruses, fungi, and archaea, this complex community resides primarily in the large intestine [4]. Microbial cells are estimated to outnumber human cells by a factor of ten, and their collective genome, the microbiome, encodes nearly 100 times more genes than the human genome [5,6].

Through a symbiotic relationship with the host, gut microbes perform numerous physiological functions that are essential for homeostasis [7,8]. These functions include the fermentation of indigestible polysaccharides and the production of short-chain fatty acids [5], enhancement of nutrient absorption such as calcium and magnesium [9], and synthesis of key vitamins including vitamin K and B-group vitamins [5,9]. The GM also contributes to immune system development by stimulating the maturation of gut-associated lymphoid tissue [9], and it influences brain function via the gut–brain axis [10].

The development of GM is particularly sensitive to early-life exposures. Critical factors such as delivery mode, feeding practices, antibiotic exposure, and environmental conditions play major roles in shaping the microbial composition. This early microbial development occurs predominantly during the first three years of life, a crucial window for immune and metabolic maturation [11]. Vaginal delivery, for example, allows for vertical microbial transmission from the mother, whereas C-section delivery is associated with altered initial colonization [9]. Breastfeeding promotes the growth of *Bifidobacteria*, while formula feeding leads to greater microbial diversity but less dominance of beneficial taxa [9]. The introduction of solid foods further drives the maturation of the microbiota [9], whereas early antibiotic exposure disrupts colonization patterns and may have long-term consequences [9,12]. Environmental factors, such as hygiene level, living in urban versus rural areas, and exposure to pets, also significantly influence microbial diversity [13]. Notably, recent evidence suggests that colonization may begin even before birth, with maternal and placental microbiomes potentially contributing to initial neonatal gut seeding [11,14].

Dysbiosis refers to an imbalance in the GM, characterized by reduced microbial diversity and pathogenic overgrowth, often caused by poor diet, antibiotics, stress, or chronic inflammation. This imbalance promotes metabolic dysfunction, systemic inflammation, and impaired intestinal barrier function [7].

As individuals age, a progressive decline in microbial diversity and ecosystem stability further disrupts host homeostasis, contributing to a wide range of metabolic, cardiovascular, inflammatory, and neurodevelopmental disorders [15]. Among these conditions, obesity is among the conditions most closely associated with microbial imbalance. Alterations in GM composition have been implicated in metabolic dysregulation [10], and individuals with obesity often present an increased *Firmicutes-to-Bacteroidetes* ratio, which may enhance energy harvest from the diet [16,17], although this association remains subject to ongoing scientific debate. Moreover, gut microbes can modulate lipid metabolism through regulation of host gene expression [18], and dysbiosis may increase intestinal permeability and trigger chronic low-grade inflammation, factors that further promote obesity and metabolic dysfunction [19].

One of the major consequences of dysbiosis is the onset of low-grade systemic inflammation, a persistent, subclinical immune activation that contributes to the development of metabolic diseases such as obesity, type 2 diabetes, and metabolic syndrome [20]. This condition is characterized by modest elevations in inflammatory markers such as TNF-α, IL-6, and CRP, and is often triggered by increased intestinal permeability, which allows bacterial products like lipopolysaccharides (LPSs) to enter the systemic circulation, activate Toll-like receptors 4 (TLR4s), and stimulate immune responses [21].

Metabolic status refers to the integrated state of physiological processes governing energy expenditure, fat accumulation, and glucose metabolism. In obesity, metabolic processes are often disrupted by chronic inflammation, insulin resistance, and disruptions in metabolic hormones. Changes in the GM can influence these processes through the production of metabolites (e.g., short-chain fatty acids—SCFAs), regulation of nutrient absorption, and modulation of the gut–liver axis [22].

In pediatric populations, specific microbial signatures have been associated with overweightness and obesity, emphasizing the profound influence of early-life microbiota on long-term health trajectories [23,24]. Fortunately, advances in sequencing technologies now allow for high-resolution profiling of the GM, opening promising avenues for the development of personalized, microbiota-targeted interventions [5,9].

Given their potential to reverse dysbiosis and modulate metabolic pathways, synbiotics have gained growing interest as a targeted therapeutic option. Synbiotics are nutritional supplements combining probiotics (beneficial live microorganisms) with prebiotics (fermentable substrates that nourish probiotics), acting synergistically to enhance GM composition, reduce inflammation, and improve metabolic markers, including insulin resistance and serum lipid profiles [21,25].

This review aims to synthesize current evidence on the role of the GM in childhood and adolescent obesity, elucidate the mechanisms by which microbial dysbiosis contributes to metabolic dysfunction and low-grade inflammation, and evaluate the potential of microbiota-modulating strategies, such as the Mediterranean diet (MD) and synbiotic supplementation, in prevention and clinical management.

## 2. Materials and Methods

A.M.K. and M.O.S., under the supervision of C.O.M., conducted a systematic literature search across PubMed, Scopus, and Web of Science, targeting studies published from January 2010 onwards that explored the relationship between gut microbiota, dietary patterns, low-grade inflammation, metabolic status, and obesity in pediatric populations. Particular emphasis was placed on studies assessing inflammatory and metabolic parameters in relation to gut microbial composition. The search strategy included keywords such as gut microbiota, dysbiosis, pediatric obesity, low-grade inflammation, metabolic status, probiotics, and diet (Table 1).

Eligible studies were full-text articles published in English, including Randomized Controlled Trials (RCTs), cohort studies, cross-sectional studies, and longitudinal studies examining the gut microbiota in relation to obesity, inflammation, and metabolism in individuals aged ≤18 years. Studies were excluded if they were case reports, editorials, narrative reviews, meta-analyses, non-English publications, abstract-only entries, or duplicates.

All sixteen included studies were critically appraised using the NIH Study Quality Assessment Tool [26] (Table 2), applying the checklist appropriate to each study design (cross-sectional, cohort, or RCT). Each item was scored as ‘Yes’, ‘No’, ‘Not Reported’ (NR), ‘Not Applicable’ (NA), or ‘Cannot Determine’ (CD). Studies receiving at least eleven ‘Yes’ responses (out of 13 or 14 items, depending on the checklist used) and demonstrating no major methodological concerns were classified as low risk of bias. All included studies fulfilled these criteria, resulting in a consistently high level of methodological rigor. No study was rated as moderate or high risk. Overall, sixteen studies were included in the final synthesis, among which four were randomized controlled trials, further strengthening the reliability of the findings. Although minor limitations inherent to observational designs were noted, they did not significantly impact the interpretation of the results.

## 3. Results

**Figure 1:**
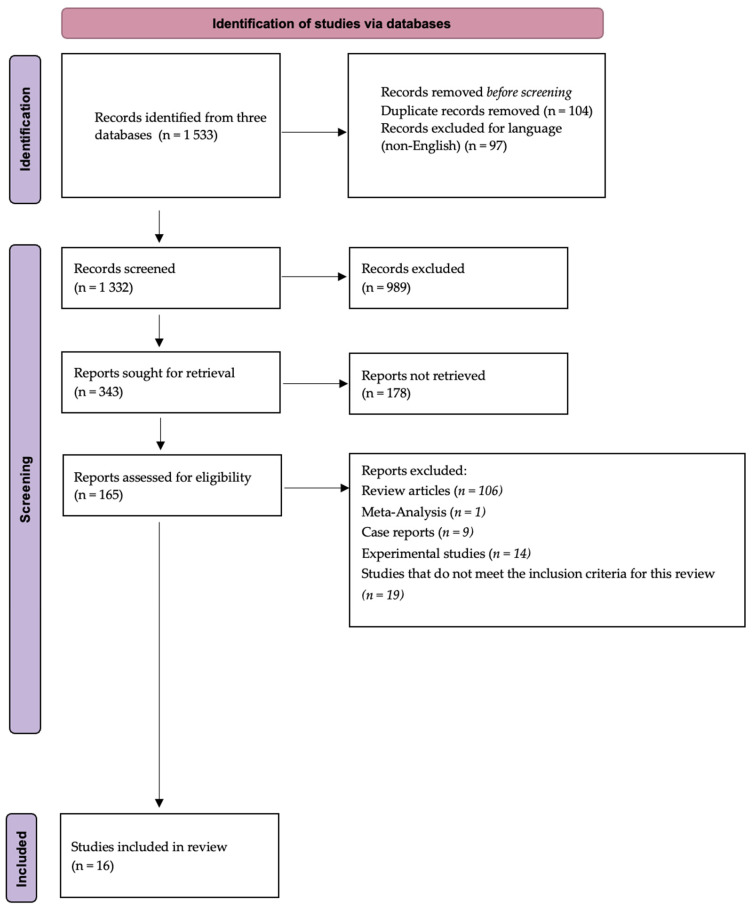
PRISMA 2020 flow diagram summarizing the selection process for studies included in the systematic review. The figure outlines the identification, screening, and eligibility assessment of studies investigating the association between gut microbiota, pediatric obesity, inflammation, and metabolic outcomes. Following predefined inclusion and exclusion criteria, 16 high-quality studies were retained for qualitative synthesis.

The initial search retrieved 1533 articles. After removing duplicates and non-English publications, 1332 titles and abstracts were screened. Of these, 989 were excluded for being irrelevant to the review topic, for example, studies focusing on unrelated conditions or lacking microbiota data. In the next phase, full-text articles were assessed, and additional studies were excluded due to unsuitable designs (e.g., experimental or in vitro studies), being meta-analyses or narrative reviews, or not reporting relevant outcomes such as inflammatory markers, metabolic parameters, or microbiota composition in pediatric obesity. Ultimately, 16 studies met all eligibility criteria and were included in the final synthesis (Figure 1, Table 3).

### 3.1. Gut Microbiota and Obesity

Gut microbiota plays a critical role in regulating host energy balance and adiposity [43]. However, how microbial diversity relates to obesity remains a subject of ongoing debate [44,45], whereas others have observed increased bacterial counts [46,47]. One of the most frequently studied microbial metrics, the *Firmicutes*-to-*Bacteroidetes* ratio, has yielded inconsistent results; while some research has found elevated ratios in obese individuals [48], others have failed to confirm any consistent association [48]. These discrepancies highlight the necessity of accounting for confounding factors such as host genetics and microbial gene richness in future investigations.

While early research emphasized phylum-level differences, more recent findings have expanded the scope to include alterations at finer taxonomic levels. More detailed analyses have revealed shifts at the family, genus, and even species levels. Families such as *Rikenellaceae*, *Ruminococcaceae*, and *Veillonellaceae* are found in lower abundance in individuals with obesity [49], while genera like *Alistipes*, *Coprococcus*, *Fusobacterium*, *Lactobacillus*, and *Bacteroides* are more prevalent [13,50,51]. Conversely, genera such as *Desulfovibrio*, *Lachnoanaerobaculum*, *Faecalibacterium*, and *Bifidobacterium animalis* are reduced in obese individuals [50,51,52]. At the species level, reductions in *Akkermansia muciniphila*, *Clostridium perfringens*, and *Bifidobacterium longum* have been consistently observed in individuals with obesity [50,52]. These findings underscore the complexity of microbial shifts in obesity. Rather than following broad phylum-level trends, microbial alterations vary considerably across taxonomic groups.

Dysbiosis, or the imbalance in gut microbial composition, has been mechanistically linked to obesity through several pathways. These include increased production of SCFAs, which influence satiety and fat storage via GPR41 and GPR43 receptors, suppression of fasting-induced adipose factor (FIAF), and improved energy harvest from food by microbes such as *Methanobrevibacter smithii* [18,52,53]. Mounting evidence supports the concept of the GM as a “metabolic organ”, substantially contributing to energy homeostasis and playing a role in the pathogenesis of obesity by enhancing caloric extraction and storage [54]. Host genetics further shape microbiota composition, as indicated by higher microbial similarity among monozygotic twins [16,55] and specific taxa such as *Christensenellaceae minuta* have been associated with a protective metabolic profile [56]. Moreover, bariatric surgery has been shown to induce dynamic shifts in microbial communities, including the restoration of glutamate-fermenting bacteria, reflecting the microbiota’s adaptability in response to physiological changes [57].

The gut–brain axis (GBA), shaped by microbial, neural, endocrine, and immune pathways, plays a pivotal role in regulating appetite, metabolism, and energy balance, thereby contributing to obesity [58]. Alterations in GM have been linked not only to obesity, but also to mood and inflammatory disorders, underscoring their systemic impact [59,60]. Key mediators such as ghrelin, GLP-1, and the vagus nerve transmit microbial signals that influence satiety and energy intake [61]. In this context, interventions including probiotics, prebiotics, synbiotics, and fecal microbiota transplantation are under investigation [62]. Furthermore, gut microbes modulate host responses via SCFAs and neurotransmitters like GABA and serotonin, affecting central appetite regulation [63,64,65]. Dysbiosis may also enhance ghrelin signaling and insulin secretion, promoting weight gain [66,67]. Given this bidirectional interaction, the GBA is increasingly recognized as a promising target in obesity therapy [68,69].

From a therapeutic perspective, dietary interventions present promising strategies to modulate GM composition. Low-energy diets, probiotics, and prebiotics have shown beneficial effects in both preclinical and clinical settings [15]. Specific compounds, such as oolong tea polyphenols and galacto-oligosaccharides (GOS), have improved microbial profiles and metabolic markers in experimental studies involving rodents [70,71]. Furthermore, increasing evidence suggests that dietary fibers can reshape the GM in children with obesity, reinforcing their potential as a cornerstone of microbiome-centered therapeutic approaches [72].

### 3.2. Gut Microbiota, Pediatric Obesity, and Low-Grade Inflammation

The gastrointestinal (GI) tract hosts a dense microbial ecosystem that regulates immunity, metabolism, and epithelial barrier function [73]. Dysbiosis may promote obesity development through multiple mechanisms, including enhanced energy harvest, increased systemic inflammation via LPS translocation, and modulation of appetite regulation [22]. Fermentation of dietary fibers by colonic microbiota produces SCFAs, primarily acetate, propionate, and butyrate, which modulate glucose and lipid metabolism, support immune function, and maintain intestinal barrier integrity [20,74].

SCFAs are absorbed via passive diffusion and transporters like MCT1 and SMCT1 [75,76], and exert their effects primarily through histone deacetylase (HDAC) inhibition and G protein-coupled receptor (GPR) activation [77,78,79,80].

SCFAs exert anti-inflammatory effects mainly via FFAR2/3 and GPR109A activation, as well as HDAC inhibition [81,82]. However, under certain conditions, they may paradoxically trigger pro-inflammatory pathways, particularly via mTOR/MAPK signaling [83]. In addition to modulating immune responses and activating AMPK, SCFAs attenuate LPS-induced inflammation, inhibit colorectal tumor cell proliferation via HDAC inhibition in a concentration-dependent manner, and enhance gut barrier integrity by upregulating tight junction proteins [84,85].

Sustained SCFA production is dependent on adequate dietary fiber intake; in contrast, fiber deficiency alters microbial composition, reduces SCFA output, and weakens gut barrier integrity, thereby promoting systemic inflammation and metabolic disease [86,87].

However, when SCFA production or signaling is impaired, due to dysbiosis, low fiber intake, or metabolic stress, chronic low-grade inflammation develops, particularly impacting pediatric populations.

In obesity, adipose tissue macrophages (ATMs) shift from an anti-inflammatory M2 to a pro-inflammatory M1 phenotype, largely in response to microbial-derived LPS, via TLR4-mediated signaling [88,89]. This transition initiates “metaflammation”, a process that promotes early-onset type 2 diabetes, hypertension, and coronary artery disease [90,91,92,93].

Obesity-associated inflammation is characterized by cytokine overexpression, immune cell infiltration, and adipose tissue hypoxia [94]. M1 macrophages and CD8^+^ T cells infiltrate visceral adipose tissue, forming crown-like structures, while peripheral monocytes and effector-memory T cells amplify inflammation [95,96].

Following immune infiltration, a chronic inflammatory state develops, marked by elevated hs-CRP and IL-6 levels, both of which correlate with central adiposity and cardiometabolic risk [97]. In severe obesity, adipose tissue can constitute up to 50% of body mass and acts as a major inflammatory reservoir, with macrophages and T cells sustaining visceral inflammation via cytokine secretion [98,99]. Leptin amplifies Th1-mediated responses [100], and although early T cell depletion can improve insulin sensitivity, systemic inflammation often persists [101]. Additionally, peripheral monocytes contribute to metabolic inflammation by releasing IL-6 and TNF-α [102]. Anti-inflammatory interventions, such as dietary n-3 polyunsaturated fatty acids (PUFAs) or agents like rosiglitazone, have shown potential in lowering NF-κB activation and pro-inflammatory cytokines [103,104]. Clinically, this chronic low-grade inflammation is frequently observed in children with metabolically unhealthy obesity (MUO), particularly those with sedentary behavior, excess caloric intake, and risk factors like post-pubertal BMI-SDS and thyroid dysfunction [105,106]. In contrast, children with metabolically healthy obesity (MHO) tend to exhibit a milder inflammatory profile, though liver steatosis is frequently present [105]. Early interventions, such as omega-3 supplementation, physical activity, and cytokine-targeted therapies, are essential for preventing progression [107].

Lifestyle factors, particularly high-fat diets and sedentary behavior, exacerbate the inflammatory state by increasing plasma LPS levels and impairing gut barrier integrity, thereby promoting metabolic endotoxemia [19,108,109]. Dysbiosis of the GM has also been linked to neuroinflammatory and neurodegenerative processes via disruption of the gut–brain axis [108]. In pediatric obesity, activation of the NLRP3 inflammasome promotes cytokine secretion, further amplifying systemic inflammation [110]. Moreover, amyloid-producing bacteria such as Escherichia coli may contribute to neurodegeneration through TLR2-mediated signaling pathways [111].

This intricate interplay between GM, inflammation, and systemic disease risk has prompted numerous studies investigating microbiota alterations in pediatric obesity. Table 4 summarizes the key findings.

### 3.3. The Mediterranean Diet: Impact on Obesity, Inflammation, and Gut Microbiota

The global rise in obesity poses a significant public health challenge, particularly in industrialized societies, where prevalence continues to increase. It is closely associated with cardiovascular disease, type 2 diabetes, and several cancers [115,116]. In response, dietary strategies have gained increasing attention not only for weight management but also for their influence on the GM, a pivotal regulator of host metabolism, immune function, and inflammation [16].

Among the patterns studied, the MD stands out. It emphasizes vegetables, fruits, legumes, whole grains, and olive oil, with moderate fish and poultry intake and limited red and processed meat. Adherence to the MD has been linked to improved metabolic health, lower chronic inflammation, and a reduced incidence of major cardiovascular events [117,118].

Notably, despite high obesity rates in Mediterranean regions, cardiovascular mortality is relatively low, suggesting that diet quality may offset metabolic risks independently of body weight [119].

Consistently, the MD’s anti-inflammatory effects are well supported by randomized controlled trials and meta-analyses, showing significant reductions in markers such as TNF-α, IL-6, and CRP [120,121].

However, evidence regarding its role in obesity prevention is mixed. Some studies report that higher adherence is associated with reduced weight gain and lower obesity risk [122,123], while a recent meta-analysis confirmed a consistent benefit over a five-year period [119].

These effects are largely attributed to the diet’s high fiber content and its ability to enhance satiety and regulate appetite through improved glycemic control, thereby promoting better weight management and overall metabolic health [124,125].

Beyond its general metabolic effects, the Mediterranean diet exerts additional benefits by modulating the GM, a complex and individualized ecosystem central to host homeostasis [126,127]. It facilitates energy extraction, regulates immune responses, and promotes the production of SCFAs, which exert critical metabolic and anti-inflammatory functions [128]. Disruption of the gut microbiota, a state known as dysbiosis, has been linked to obesity and related metabolic condi-tions [129].

Dietary fiber, a hallmark of the MD, serves as a primary substrate for beneficial gut microbes, stimulating the production of short-chain fatty acids, particularly propionate, that regulate appetite and support metabolic health [130,131]. These microbial metabolites have been shown to improve insulin sensitivity, reduce inflammation, and modulate appetite regulation [132].

Consistent with this, studies have demonstrated that individuals adhering to the MD exhibit higher levels of SCFA-producing bacteria, such as *Faecalibacterium prausnitzii*, *Akkermansia muciniphila*, and *Christensenellaceae*, alongside a lower *Firmicutes*/*Bacteroidetes* ratio [133,134].

Altogether, these insights emphasize the importance of dietary interventions targeting the GM as promising strategies for the prevention and management of obesity and its associated metabolic complications.

### 3.4. Synbiotics, Probiotics, and Prebiotics in Obesity

Synbiotics are defined as “mixtures of probiotics and prebiotics that beneficially affect the host by promoting the survival, implantation, and activity of health-promoting microorganisms” [135], with current consensus emphasizing that the substrate must be selectively utilized by host microbes and confer a demonstrable health benefit [136]. Synbiotics can be classified as complementary, where each component meets established probiotic or prebiotic criteria and acts independently, or synergistic, wherein the substrate is designed to be selectively utilized by the co-administered microorganism [135]. Despite their potential, most commercial products are complementary, as synergistic designs are more complex to validate [137].

For a product to be legitimately labeled a synbiotic, it must demonstrate both a measurable health benefit and selective substrate utilization, whether by endogenous microbiota or the administered strain [138]. Ensuring the quality and reliability of probiotic products requires rigorous characterization, including whole genome sequencing for precise strain identification, comprehensive safety validation in accordance with established guidelines, and thorough assessments of product purity and stability [139]. Human studies must adhere to CONSORT guidelines and be adequately powered to substantiate efficacy claims [140,141].

Obesity, a multifactorial disease, has been associated with gut dysbiosis, characterized by an increased Firmicutes-to-Bacteroidetes ratio and alterations in microbial diversity [142,143]. Emerging evidence suggests that synbiotic interventions may help restore microbial balance and metabolic health by promoting mucin and antimicrobial peptide production, strengthening tight junction integrity, modulating immune responses, and suppressing pathogen adherence [144]. Although their impact can occur without permanent colonization, more evidence is required to substantiate their therapeutic roles, particularly in immunocompromised populations [145,146].

To extend the mechanistic insights discussed above, Table 5 summarizes recent clinical studies evaluating the effects of probiotic, synbiotic, and dietary interventions on GM composition and metabolic outcomes in pediatric obesity.

These findings highlight the therapeutic potential of targeted probiotic and synbiotic interventions in restoring gut homeostasis and improving metabolic and immune health. Nonetheless, further longitudinal studies are warranted to validate their long-term efficacy and safety profiles.

## 4. Discussion

### 4.1. Gut Microbiota Dysbiosis and Pediatric Obesity

The GM has emerged as a crucial mediator linking pediatric obesity with systemic inflammation, metabolic dysfunction, and impaired energy balance [37,38]. Studies consistently report a reduction in microbial diversity among obese children, characterized by a depletion of beneficial taxa such as *Faecalibacterium prausnitzii* and *Akkermansia muciniphila*, alongside an expansion of pro-inflammatory genera like *Streptococcus* and *Desulfovibrio* [112,113]. These alterations suggest that microbial dysbiosis drives low-grade systemic inflammation and adiposity, as supported by both mechanistic and human studies [16,108,149].

This dysbiotic shift is believed to enhance intestinal permeability, allowing bacterial LPS to enter systemic circulation and trigger chronic inflammation, thus promoting insulin resistance and hepatic steatosis [32,39]. Additionally, the enrichment of specific microbes like *Methanobrevibacter smithii*, associated with organic dairy consumption, may enhance intestinal fermentation efficiency, potentially contributing to an obesogenic phenotype [30].

However, the directionality of the relationship between gut dysbiosis and pediatric obesity remains uncertain and is likely bidirectional. While controlled experimental studies and some longitudinal human cohorts have demonstrated that alterations in gut microbiota can precede and contribute to the development of obesity-related phenotypes, particularly through fecal microbiota transplantation into microbiota-depleted hosts, current evidence in pediatric populations remains largely observational and insufficient to establish causality [27,150].

It is equally plausible that obesity-related dietary patterns, host metabolism, and low-grade inflammation have also been shown to influence gut microbial composition [150]. In pediatric populations, causality is especially difficult to establish due to confounders such as antibiotic exposure, delivery mode, age-related microbial maturation, and dietary variability. Even when bacterial taxa are associated with metabolic markers (e.g., *Bifidobacterium longum*, *Faecalibacterium prausnitzii*), these relationships may reflect underlying dietary patterns rather than direct microbial effects [31,38,151].

Thus, despite promising associations, the GM–obesity relationship in children must be interpreted with caution. Robust prospective cohorts, longitudinal microbiota tracking, and randomized, microbiota-targeted dietary interventions are essential to delineate causal mechanisms and support translation into pediatric clinical strategies [39,150].

### 4.2. Low-Grade Inflammation and Gut Microbiota in Pediatric Obesity

Low-grade systemic inflammation is a hallmark of MUO in children and adolescents, increasingly recognized as a contributor to metabolic dysfunction [39,112]. It is typically subclinical and chronic, involving immune and metabolic disturbances even in the absence of symptoms [112]. This immune dysregulation has been linked to alterations in GM composition [39].

Mechanistically, gut dysbiosis contributes to inflammation by impairing intestinal barrier function and facilitating the translocation of microbial products, such as LPS, into systemic circulation. This activates TLR4-mediated immune responses [38,129]. The resulting process, known as *“metabolic endotoxemia”*, triggers the activation of innate immunity and sustains pro-inflammatory signaling associated with insulin resistance [113,129].

Several studies support associations between specific microbial shifts and heightened inflammatory markers. For instance, Del Chierico et al. reported that obese children exhibited increased *Ruminococcus* and *Bacteroides caccae*, along with reduced *Faecalibacterium prausnitzii* and *Akkermansia muciniphila*, a pattern linked to elevated white blood cell counts and adverse metabolic profiles [112]. Similarly, Squilliario et al. found higher levels of *Streptococcus* and *Acidaminococcus*, and reduced *Bifidobacterium longum* and *Alistipes*, strongly correlated with CRP and LDL cholesterol [113]. Zhang et al. reinforced these findings by reporting elevated *Clostridium* and *Escherichia coli*, along with increased LPS, implicating Gram-negative bacterial overgrowth in systemic immune activation [38].

Altered SCFA metabolism, particularly lower fecal acetate and propionate, has also been observed in children with stunted growth [114]. These reductions may impair gut barrier integrity, potentially contributing to a low-grade inflammatory state [114,129]. Although these children were not obese, the findings highlight the broader role of microbial metabolic activity, suggesting that inflammation may arise from functional rather than compositional dysbiosis [129].

Despite accumulating evidence, the causal direction remains unclear. Some authors propose that inflammation may precede and drive microbiota shifts, especially in the context of early-life exposures, diet, or genetic predisposition [129]. Others suggest a reverse pathway, where dysbiosis and its metabolic byproducts initiate immune activation [112]. Additionally, González-Domínguez et al. argue that metabolic inflexibility, rather than inflammation per se, may mediate the link between gut microbial composition and insulin resistance [39].

Microbial signatures also appear to vary with nutritional and physiological status [112,114]. While some children show clear signs of inflammation and microbial imbalance, others, despite growth impairments, maintain microbial diversity and gut barrier function [114]. This heterogeneity implies that immune activation is not uniform and may be modulated by factors such as age, pubertal development, environmental exposures, and diet [112].

Taken together, current evidence underscores a strong, albeit complex, relationship between GM and low-grade inflammation in pediatric obesity. The precise mechanisms and directionality of this interplay remain to be clarified. Further longitudinal and interventional studies will be essential to clarify causal links and guide microbiota-targeted prevention and treatment strategies.

### 4.3. Dietary Interventions and Microbiota Modulation

High-fiber diets have been shown to beneficially modulate GM composition, promoting the growth of functional taxa such as *Bifidobacterium longum* and *Faecalibacterium prausnitzii*, which are known SCFA-producing bacteria with roles in metabolic regulation [31]. These microbial shifts are associated with improved glycemic control, reduced inflammation, and favorable changes in body composition in children, as demonstrated in recent clinical trials [37,152].

Although short-term interventions show promising microbial shifts, the long-term stability of these effects beyond the dietary phase remains unclear [38]. This highlights the importance of long-term adherence to fiber-rich eating patterns, as short-term interventions may induce temporary microbial changes that quickly revert after dietary discontinuation [153].

The MD, characterized by high intake of vegetables, fruits, legumes, whole grains, and olive oil, has emerged as a microbiota-supportive dietary strategy. Adherence to the MD is associated with increased microbial diversity and higher abundance of taxa linked to metabolic health, such as *Faecalibacterium prausnitzii* and *Roseburia* spp. [153]. These microbial changes are paralleled by reductions in inflammatory markers and improvements in insulin sensitivity, as demonstrated in pediatric trials using probiotic or prebiotic supplementation [37,152].

However, the adoption of the MD in non-Mediterranean pediatric populations is limited by cultural preferences, food availability, and socioeconomic factors [154]. Such barriers may limit the practical implementation of this approach, especially in low-resource settings.

SCFAs, in particular, act as key mediators at the intersection of microbiota, diet, and host metabolism [74]. While their overall effects are beneficial, especially when fiber intake is adequate, their impact must be interpreted in the broader context of dietary patterns and host physiology [31,155].

In conclusion, dietary interventions represent a cornerstone of microbiota-targeted strategies in pediatric obesity. High-fiber, plant-based diets and Mediterranean-style patterns promote favorable microbial configurations and improve metabolic outcomes [31]. However, their clinical utility depends not only on microbiota responsiveness but also on long-term dietary adherence, cultural feasibility, and personalized nutritional guidance [38].

### 4.4. Synbiotics, Prebiotics, and Probiotics

Probiotic supplementation has attracted growing attention as a potential adjunct therapy for pediatric obesity through its ability to influence host metabolism and immune function [37,42,155]. Strains such as *Bifidobacterium breve* BR03 and B632, and *Bifidobacterium animalis* subsp. *lactis* BPL1 have demonstrated metabolic improvements including enhanced insulin sensitivity, reduced waist circumference, and decreased inflammatory markers in clinical trials involving obese children [37,42].

While clinical outcomes are encouraging, probiotics tend to induce modest changes in overall GM structure [29,37]. This suggests that their benefits arise from targeted host–microbe signaling or metabolite production, rather than broad ecological shifts [37,86].

One proposed mechanism involves microbial components such as lipoteichoic acid, which may modulate host signaling pathways via receptor interaction, contributing to metabolic and immune effects [37]. Additionally, probiotic-induced shifts in bile acid metabolism and modulation of gut hormone secretion (e.g., GLP-1, leptin) may also contribute to their metabolic benefits [87].

Importantly, the effectiveness of probiotics appears to be strain-specific, dose-dependent, and influenced by the baseline microbiota of the host [155]. These variables underscore the need for tailored interventions rather than generalized supplementation approaches.

In summary, probiotics offer targeted metabolic benefits in pediatric obesity, but their effects on overall microbial ecology remain limited [29,37]. To address these limitations and potentially amplify therapeutic efficacy, research has increasingly turned toward synbiotic approaches, formulations that combine probiotics with selective prebiotic substrates designed to enhance microbial viability, stimulate beneficial taxa, and modulate host metabolism and immunity, partly via SCFA production [36].

The abundance of *Bifidobacterium* and *Lactobacillus* species, often reduced in pediatric obesity, may be enhanced by targeted probiotic or prebiotic interventions [147].

These taxa contribute to the production of SCFAs, reinforce mucosal integrity, and suppress endotoxin translocation [74], and therapeutic strategies aimed at restoring SCFA-producing bacteria, through dietary fibers or synbiotic supplementation, may offer meaningful metabolic improvements in pediatric populations [36].

Compared to probiotics alone, synbiotics may provide more consistent ecological and metabolic improvements, particularly when the substrate is tailored to support the co-administered microbial strain [148]. Clinical trials using probiotic-only formulations, such as BPL1 or *B. breve* strains, suggest limited effects on overall microbial composition, highlighting the potential for more effective outcomes with synbiotic combinations designed for mechanistic synergy [37,42]. Truly synergistic synbiotics, where the substrate is selectively metabolized by the specific strain, are more complex to design and validate [136].

A clinical study in obese children suggests that synbiotic supplementation can lower inflammatory markers (e.g., TNF-α, IL-6), increase adiponectin levels, and modestly improve body composition and insulin sensitivity [148]. However, responses vary significantly depending on strain identity, duration, and baseline microbiota [147,148].

In conclusion, synbiotic strategies hold strong theoretical and preliminary clinical promise in pediatric obesity. Effective synbiotic strategies require precise strain–substrate pairing, mechanistic validation, and sustained adherence [36,136]. As microbiome research advances, synbiotics may emerge as a refined and sustainable strategy for restoring microbial balance and reducing long-term metabolic risks in children with obesity [36,148].

### 4.5. Persistent Limitations and Areas for Advancement

While this review offers a comprehensive synthesis of current evidence on the interplay between gut microbiota and pediatric obesity, several limitations should be acknowledged. One major concern is the considerable heterogeneity across the included studies, encompassing differences in design (e.g., cross-sectional, cohort, RCTs), participant characteristics, types of dietary or probiotic interventions, and microbiota assessment techniques (such as 16S rRNA sequencing versus metagenomics). This variability complicates cross-study comparisons and limits the generalizability of the findings.

Moreover, many studies involved small sample sizes and short intervention periods, which reduce statistical power and hinder the evaluation of sustained microbiota or metabolic effects. The predominantly observational nature of the included research also constrains causal inference, especially given the bidirectional relationship between gut microbial alterations and factors such as diet, systemic inflammation, and host metabolism.

Finally, mechanistic understanding remains limited, as few studies employed integrated multi-omic approaches (such as metabolomics, transcriptomics, or immune profiling) to elucidate how specific microbial taxa or their metabolites influence metabolic outcomes. Future investigations should prioritize standardized protocols, extended follow-up, and personalized, mechanistically informed interventions to better define the role of microbiota modulation in the prevention and management of pediatric obesity.

To fully unlock the therapeutic potential of microbiota-based interventions in pediatric obesity requires a more strategic and personalized approach. Several priorities should guide future research:

(1) Standardized, long-term clinical trials: Many existing studies are short in duration and lack methodological consistency. Future trials should use harmonized protocols, standardizing intervention length, strain selection, and outcome measures, and follow participants long enough to track sustained effects. Well-designed randomized controlled trials will be key to building reliable, comparable evidence.
(2) Multi-omic integration and mechanistic clarity: Understanding how microbial changes drive metabolic improvements is essential. Studies should combine metagenomics, metabolomics, transcriptomics, and immune profiling to identify causal pathways and biomarkers. For instance, decoding how SCFAs or bile acids influence inflammation and fat storage could lead to more targeted interventions.
(3) Personalized interventions: Microbiota composition and host genetics vary widely between individuals, so blanket strategies will not work. Future therapies should be tailored based on microbial signatures, genetic markers (such as AMY1 copy number), and environmental context. Machine learning could help predict responses and match patients with the most effective treatments.
(4) New therapeutic tools: Beyond traditional probiotics, next-generation solutions, like engineered microbes, synbiotics with strain-matched substrates, postbiotics, and bacteriophage therapies, offer exciting new options. Such approaches may be particularly beneficial for metabolically vulnerable pediatric populations. Translating microbiota-targeted therapies into clinical care requires well-designed RCTs with standardized protocols and long-term follow-up in pediatric populations.

In short, the future lies in personalized, integrative, and mechanistically driven science. Advancing this field will depend on collaborative efforts across disciplines to develop microbiota-based interventions that are not only clinically effective but also practical and adaptable across diverse pediatric populations.

### 4.6. Clinical Implications and Translational Outlook

While further longitudinal validation is warranted, the current body of evidence provides several translational opportunities for interim clinical guidance. Dietary patterns rich in fiber, particularly Mediterranean-style diets, may be strategically recommended to support gut microbial diversity, mitigate low-grade systemic inflammation, and promote metabolic homeostasis in pediatric populations.

In addition, supplementation with specific probiotic or synbiotic formulations, such as those containing *Bifidobacterium breve* or *B. animalis* subsp. *lactis*, has demonstrated potential to improve insulin sensitivity and modulate adiposity-related markers, and may be cautiously integrated into individualized management plans.

From a preventive standpoint, early-life exposure remains critical. Promoting vaginal delivery, breastfeeding, and the prudent use of antibiotics during infancy may facilitate the establishment of a favorable gut microbial environment. Moreover, incorporating inflammatory and microbial markers into risk stratification models could enhance the early detection of MUO, pending further biomarker standardization.

Collectively, these findings underscore the clinical utility of microbiota-informed dietary and lifestyle interventions as adjunctive strategies in the management of pediatric obesity, even prior to the establishment of fully personalized approaches.

## 5. Conclusions

The GM plays a central role in pediatric obesity, acting as a key link between diet, immunity, and metabolism. Disruptions in microbial composition, marked by reduced diversity, loss of SCFA-producing bacteria, and expansion of pro-inflammatory species, are consistently associated with low-grade inflammation, insulin resistance, and fat accumulation in children.

Targeted strategies such as high-fiber diets, Mediterranean-style eating, and selected probiotic or synbiotic formulations hold real promise for modulating metabolic and immune responses. Yet their clinical impact remains variable, shaped by individual host factors, microbial context, and differences in study design. Moreover, challenges like inconsistent methodologies, short follow-up periods, and limited mechanistic insight continue to limit broader application.

Personalized interventions rooted in mechanistic understanding and supported by long-term adherence are essential for addressing pediatric obesity. Restoring gut microbial balance through diet and targeted supplementation shows promise but requires robust, integrative strategies that combine clinical, nutritional, and microbiome expertise.

## Figures and Tables

**Table 1:** Search strategy.

Component	Details
Keywords	“gut microbiota” OR “dysbiosis” AND “pediatric obesity” OR “childhood obesity” AND “low-grade inflammation” AND “metabolic status” AND “synbiotics”
Databases Searched	PubMed, Scopus, Web of Science
Database-Specific Search Details	PubMed: (“gut microbiota” OR “dysbiosis”) AND “childhood obesity” AND “low-grade inflammation” Scopus: (“gut microbiota” AND “pediatric obesity”) AND (“inflammation” OR “metabolism”) Web of Science: (“gut microbiota” AND “children”) AND (“obesity” AND “inflammatory markers” OR “metabolic profile”)
Timeframe	January 2010–Present
Language	English only
Study Types Included	RCT, cohort studies, cross-sectional studies, longitudinal studies
Additional Sources	Reference lists of included articles and relevant systematic reviews
Inclusion Criteria	- Full-text in English - Pediatric population (≤18 years) - Studies examining gut microbiota and its relationship with obesity - Data on inflammatory or metabolic parameters - Studies evaluating dietary or synbiotic interventions
Exclusion Criteria	- Case reports - Editorials - Narrative reviews and meta-analyses - Non-English publications - Abstract-only entries - Duplicate studies
Study Categories	- Microbiota composition in obese vs. non-obese children - Gut microbiota and inflammatory/metabolic markers - Dietary interventions (e.g., Mediterranean diet) - Synbiotic/probiotic therapies

Abbreviations: RCT—Randomized Controlled Trial.

**Table 2:** Critical appraisal of included studies.

Study	Study Design	Risk of Bias (NIH)	Justification
Mbakwa et al. (2018) [27]	Cross-sectional	Low	Robust design, adjustment for confounders
Nobili et al. (2024) [28]	Cross-sectional	Low	Updated study, solid data
Rahayu et al. (2021) [29]	RCT	Low	Double-blind, placebo-controlled probiotic intervention with appropriate randomization and good control of confounders
Van de Pol et al. (2017) [30]	Cross-sectional	Low	Adequate control, diet and age adjustments
Xiang et al. (2021) [31]	Cross-sectional	Low	Well-structured microbiota analysis
Yang et al. (2014) [32]	Cross-sectional	Low	Study on pediatric NAFLD linked to microbiota
Kisuse et al. (2018) [33]	Cross-sectional	Low	Low selection bias, good confounder control
Liang et al. (2015) [34]	Cross-sectional	Low	Appropriate statistical adjustment, diverse population
Mimila et al. (2018) [35]	Cross-sectional	Low	Well-supported correlations, adjustment for confounders
Barczynska et al. (2016) [36]	Intervention study (quasi-RCT)	Low	Prebiotic intervention with partial randomization; moderate risk due to unclear blinding and allocation
Amat-Bou et al. (2020) [37]	RCT	Low	Randomized crossover design, valid measures, low attrition
Zhang et al. (2015) [38]	Intervention cohort	Low	Dietary intervention, robust multi-omics analysis
Gonzales-Dominguez et al. (2024) [39]	Mixed-method (observational + experimental)	Low	Multifaceted experimental design, prospective with metabolic outcomes
Lind et al. (2019) [40]	RCT	Low	Well-defined RCT protocol, valid tools, low loss to follow-up
Mennella et al. (2022) [41]	RCT	Low	Randomized design, valid exposure and outcome measures
Solito et al. (2021) [42]	RCT	Low	Double-blind, placebo-controlled, appropriate statistical modeling

Abbreviations: RCT—Randomized Controlled Trial; NIH—National Institutes of Health Study Quality Assessment Tool; NAFLD—Non-Alcoholic Fatty Liver Disease.

**Table 3:** Summary of included studies.

Author (Year)	Study Design	Population	Sample Size	Key Outcomes
Mbakwa et al. (2018) [27]	Cross-sectional	Children	180	GM composition associated with BMI and body fat
Nobili et al. (2024) [28]	Cross-sectional	Children with NAFLD	402	Microbiota differences linked with NAFLD severity
Rahayu et al. (2021) [29]	RCT	Obese and non-obese children	81	Probiotic intervention modified GM and improved metabolic parameters in obese children
Van de Pol et al. (2017) [30]	Cross-sectional	Overweight/obese children	150	GM altered in overweight/obese vs. normal weight
Xiang et al. (2021) [31]	Cross-sectional	Children	250	Associations between GM and metabolic risk factors
Yang et al. (2014) [32]	Cross-sectional	Children with NAFLD	59	Microbial shifts associated with pediatric NAFLD
Kisuse et al. (2018) [33]	Cross-sectional	Children	50	Gut microbial diversity differences in children with obesity
Liang et al. (2015) [34]	Cross-sectional	Children	149	Relationship between GM and anthropometric measures
Mimila et al. (2018) [35]	Cross-sectional	Mexican children	66	Microbiota differences in obese vs. normal-weight Mexican children
Barczynska et al. (2016) [36]	Intervention study (quasi-RCT)	Obese children	60	Maize dextrins selectively modulate GM in overweight and obese children
Amat-Bou et al. (2020) [37]	RCT	Children with obesity	40	Synbiotic supplementation improves obesity markers
Zhang et al. (2015) [38]	Intervention cohort	Obese adolescents	79	Diet modulates gut microbiota and metabolic parameters
Gonzales-Dominguez et al. (2024) [39]	Mixed-method (observational + experimental)	Adolescents with obesity	80	Metabolomic disturbances linked to insulin resistance in adolescents with obesity
Lind et al. (2019) [40]	ongoing RCT—expected outcomes (protocol study)	Infants	70	Formula feeding impacts microbiota and metabolism
Mennella et al. (2022) [41]	RCT	Infants	60	Formula composition affects GM development
Solito et al. (2021) [42]	RCT	Children	70	Bifidobacteria supplementation improves gut health in children

Abbreviations: BMI—Body Mass Index; GM—Gut Microbiota; RCT—Randomized Controlled Trial; NAFLD—Non-Alcoholic Fatty Liver Disease.

**Table 4:** Inflammation-related gut microbiota changes in obese children.

Author and Year	Study Type	Population	Intervention	Main Findings
Del Chierico et al., 2021 [112]	Case–control	Obese children (exact N not specified)	Microbiota and metabolome profiling	↓ *F. prausnitzii*, ↓ *A. muciniphila*, ↑ *Ruminococcus*, ↑ *B. caccae*; associated with ↑ IL-6, TNF-α; disrupted SCFA metabolism.
Squilliario et al., 2023 [113]	Cross-sectional	Obese vs. normal-weight children	Microbiota sequencing in obese children	↑ *Streptococcus*, ↓ *B. longum*; ↑ *Acidaminococcus*, ↓ *Alistipes*; correlated with IL-6, TNF-α and insulin resistance.
Surono et al., 2024 [114]	Comparative observational	100 stunted vs. 100 non-stunted children (36–45 months)	16S rRNA sequencing, SCFA analysis	↓ *Butyrivibrio*, ↓ *Roseburia*; ↓ butyrate/propionate; findings in stunted children suggest gut barrier dysfunction and inflammation mechanisms relevant to metabolic disease risk.
Zhang et al., 2015 [38]	Case–control	21 obese children	Metagenomics and inflammatory markers	↑ *Firmicutes* (*Clostridium*), ↓ *Bifidobacterium*, ↑ *E. coli* (*Enterobacteriaceae*), ↑ LPS; linked to low-grade inflammation.
González-Domínguez et al., 2024 [39]	Observational + in vivo/ex vivo	Obese ± insulin resistance, healthy controls (N not specified)	OGTT, erythrocyte metabolomics	Insulin resistance linked to impaired metabolic flexibility and inflammatory shifts; microbiota-metabolome interaction suggested.

Note: Surono et al. (2024) [114]—although the study focused on stunted children, the observed disruptions in SCFA metabolism and gut microbiota composition highlight mechanisms that may be relevant to metabolic and inflammatory pathways associated with pediatric obesity. Abbreviations: IL-6—interleukin-6; LPS—lipopolysaccharides; OGTT—oral glucose tolerance test; SCFA—short-chain fatty acids; TNF-α—tumor necrosis factor-alpha; 16S rRNA—16S ribosomal ribonucleic acid gene (used in microbial profiling); *E. coli*—*Escherichia coli*.

**Table 5:** Summary of clinical studies evaluating probiotic, synbiotic, and dietary interventions on gut microbiota and metabolic outcomes in obese children.

Author and Year	Study Type	Population	Intervention	Main Findings
Solito et al., 2021 [42]	RCT (cross-over, double-blind)	101 obese children/adolescents	*B. breve* BR03 and B632 probiotics (8 weeks)	↑ Insulin sensitivity (↑QUICKI, ↑ISI), ↓ waist circumference, ↓ fasting insulin, ↓ *E. coli*; SCFA stable in probiotics group.
Nagata et al., 2017 [147]	Open prospective study	12 obese vs. 22 healthy children	LcS probiotic for 6 months following diet/exercise	↑ *Bifidobacterium* and acetic acid, ↓ *B. fragilis*, *Atopobium cluster, L. gasseri*; ↓ weight, ↑ HDL.
Kelishadi et al., 2014 [148]	Triple-masked RCT	70 overweight/obese children (56 completed)	Synbiotics (8 weeks)	↓ TNF-α, ↓ IL-6, ↑ adiponectin; possible link to ↑ *Bifidobacterium* and *Lactobacillus* abundance (BMI-adjusted effects NS).
Mennella et al., 2022 [41]	RCT	30 infants (subset of RCT)	Cow milk vs. hydrolyzed formula	Hydrolyzed formula → ↑ *Ruminococcus gnavus* and *Clostridia*; faster microbiota maturation, ↓ weight gain.
Lind et al., 2019 [40]	Study protocol (ongoing RCT)	~250 infants (planned)	Nordic diet-based complementary feeding	Expected: ↑ *Bacteroides*, ↑ *F. prausnitzii*, ↓ *Firmicutes*; better microbiota and body composition (protocol phase).
Amat-Bou et al., 2020 [37]	Randomized crossover trial	39 children with Prader–Willi	12-week probiotic (BPL1) vs. placebo, with crossover	BPL1 reduced abdominal adiposity, improved insulin sensitivity, and mental health scores (children > 4.5 years).

Note: Lind et al. (2019) [40] describes an ongoing randomized controlled trial (study protocol), and therefore reports expected outcomes rather than finalized clinical results. Abbreviations: BMI—body mass index; *BR03* and *B632—Bifidobacterium breve strains; BPL1—Bifidobacterium animalis subsp. lactis*; *Clostridia*—a class of Firmicutes bacteria commonly found in the gut; *F. prausnitzii—Faecalibacterium prausnitzii*; HDL—high-density lipoprotein; IL-6—interleukin-6; ISI—Matsuda Insulin Sensitivity Index; *LcS—Lactobacillus casei* strain Shirota; NS—not statistically significant; SCFA—short-chain fatty acids; TNF-α—tumor necrosis factor-alpha; QUICKI—Quantitative Insulin Sensitivity Check Index; RCT—randomized controlled trial; *Ruminococcus gnavus*—a gut commensal bacterium associated with mucin degradation.
